# Response to: Medical Ethics According to Avicenna’s Stance: A Synopsis

**DOI:** 10.31661/gmj.v12i.2794

**Published:** 2023-06-10

**Authors:** Hamed Arezaei, Majid Dadmehr

**Affiliations:** ^1^ Department of Traditional Medicine, School of Persian Medicine, Iran University of Medical Sciences, Tehran, Iran; ^2^ Department of Traditional Medicine, Institute for Studies in Medical History, Persian and Complementary Medicine, Iran University of Medical Sciences, Tehran, Iran

**Keywords:** Medical Ethics, History of Medicine, Avicenna, Quṭb al-Din

## Dear editor,

We read the article "Medical Ethics According to Avicenna’s Stance: A Synopsis" with great interest [1]. According to the authors, Avicenna’s recommendations on the medical ethics are stated in the third chapter of the Fī Bayān al-Ḥājah ila al-Ṭibb wa al-Aṭibbā wa Waṣāyāhum written by Quṭb al-Dīn Shīrāzī (1236-1311 AD), who was the main commentator of the Canon of Medicine. The authors have also mentioned that Avicenna has advised physicians to consider several ethical issues, including the patient’s interests, communication skills, and adherence to the characteristics of professional excellence in their practice and dealing with patients [1]. A precise review of the Canon of Medicine shows that this is a misinterpretation of Avicenna’s own opinion [2]. Therefore, we suggest some more details to clarify this subject that can be useful and complementary.

A brief overview about Quṭb al-Dīn Shīrāzī (1236-1311 AD) and his works

Quṭb al-Dīn Shīrāzī (also known as Allāma Shīrāzī) was a prominent Persian polymath who has written many books in various fields of science, including medical science. He authored his well-known textbook on medicine al-Tuḥfa al-Saʿdīya, as a comprehensive commentary on the first volume of the Canon of Avicenna [3][4].

Quṭb al-Dīn was writing this book until the last year of his life (1311 AD), and when he passed away, some parts of the book were not yet finished. He had reviewed other commentaries on the Canon of Medicine that were written before him and criticized them in his work. In addition to collecting the different viewpoints of his predecessors, Quṭb al-Dīn discussed carefully the subjects based on his wide views and knowledge of different branches of science. Therefore, his commentary is an encyclopedia that covers various medical and medical-related topics in addition to explanations about Avicenna’s opinions [3][5].

The encyclopedic nature of this book has caused Quṭb al-Dīn to occasionally include topics that are not merely explanations of the terms in the Canon of Medicine. The description of several medical ethics issues in the al-Tuḥfa al-Saʿdīya is considered one of these examples.

In this article, we reviewed the original versions of the al-Tuḥfa al-Saʿdīya, which contain its whole volumes. The list of them are as follows:

- MSS 2047-2050, copied in 1318 AD, kept in the Şehid ʿAli Paşa library, Istanbul, Turkey [6].

- MSS 3649-3656, copied in 1336 AD, kept in the Ayasofya library, Istanbul, Turkey [7].

- MS 3904, copied in 1490 AD, kept in the Majlis Shura library, Tehran, Iran [8].

The Medical Ethics in the Canon of Medicine

The Canon of Medicine consists of five volumes. Its first volume is called al-Kullīyyāt, which contains principles of medicine, including general anatomy, physiology, etiology and health, and symptomology [2][5]. Although Ibn Sīnā (also known as Avicenna) presented a chapter called "waṣīyyah" at the end of the first volume, he did not dedicate any specific chapter to the medical ethics. The word "waṣīyyah" can mean both moral advice and professional teachings. According to the content of this part in the Canon of Medicine, Ibn Sīnā used the word in the second meaning [2]. Therefore, Quṭb al-Dīn used this opportunity and explained his personal opinions along with the viewpoints of his predecessors on the ethical issues under this title as a commentator.

About the Fī Bayān al-Ḥājah ila al-Ṭibb wa al-Aṭibbā wa Waṣāyāhum

In the different parts of the al-Tuḥfa al-Saʿdīya, the medical ethics issues are discussed in the form of various topics, such as professional recommendations, talking about death, teaching methods of medical knowledge, etc. [6][7][8]. Contrary to the opinion of the authors [1], Quṭb al-Dīn did not have a distinct book or treatise in the field of medical ethics. The bibliographic evaluation of the manuscripts indicates that in order to provide a separate book on the medical ethics, some scholars interested in ethical issues who lived after Quṭb al-Dīn tried to collect these pages related to medical ethics. They have selected and transcribed relevant parts of the al-Tuḥfa al-Saʿdīya just to teach their students in the form of a single book named Fī Bayān al-Ḥājah ila al-Ṭibb wa al-Aṭibbā wa Waṣāyāhum. Therefore, these contents were not compiled by Quṭb al-Dīn himself in the form of a specific book on medical ethics. This can be seen in the few remaining manuscripts of the medical ethics based on the al-Tuḥfa al-Saʿdīya, edited by al-Dhā kirī and al-Mizyadī [9][10]. Reviewing and comparing the expressions used in different parts of these manuscripts with the original versions of the al-Tuḥfa al-Saʿdīya, clearly proves that the main content is related to the al-Tuḥfa al-Saʿdīya by Quṭb al-Dīn [6][7][8][9][10].

Although Muslim scholars were concerned with medical ethics, its separation from the discipline of medicine as a distinct branch of science happened later. Ibn Sīnā considered the medical ethics to be outside the scope of medicine, therefore, even though he authored some contents on ethics, he did not dedicate any specific chapter to medical ethics in the Canon of Medicine. In the commentary of the first volume of the Canon, Quṭb al-Dīn devoted scattered parts of the al-Tuḥfa al-Saʿdīya to the topics of medical ethics. In order to highlight medical ethics as a specific field and to make the medical community of their time pay attention to ethical issues, the scholars who lived after Quṭb al-Dīn created works from the scattered writings of the predecessors’ books to fill the void of independent books in the field of medical ethics.

## Conflict of Interest

The authors declare that they have no conflict of interest.
